# Regulatory landscape for new breeding techniques (NBTs): insights from Paraguay

**DOI:** 10.3389/fbioe.2024.1332851

**Published:** 2024-01-24

**Authors:** Danilo Fernández Ríos, Nidia Benítez Candia, María Cristina Soerensen, María Florencia Goberna, Andrea Alejandra Arrúa

**Affiliations:** ^1^ Departamento de Biotecnología, Facultad de Ciencias Exactas y Naturales, Universidad Nacional de Asunción, San Lorenzo, Paraguay; ^2^ Dirección de Comercio Internacional, Dirección General de Planificación, Ministerio de Agricultura y Ganadería, Asunción, Paraguay; ^3^ Coordination of Innovation and Biotechnology, National Directorate of Bioeconomy, Under-Secretariat of Food, Bioeconomy and Regional Development, SAGyP, Buenos Aires, Argentina; ^4^ Centro Multidisciplinario de Investigaciones Tecnológicas, Universidad Nacional de Asunción, San Lorenzo, Paraguay

**Keywords:** Paraguay, regulatory framework, new breeding techniques, genome editing, biotechnology

## Introduction

Regulation of New Breeding Techniques (NBTs)^1^ in agriculture has garnered considerable attention and discussion in recent years. These emerging technologies have the potential to greatly impact crop productivity and address a range of global challenges, prompting countries worldwide to grapple with how best to regulate and oversee their use.

Despite the abundance of scholarly literature discussing the regulatory dimensions of these biotechnological advancements (Menz et al., 2020; Entine et al., 2021; Turnbull et al., 2021; Rosado and Eriksson, 2022; Sprink et al., 2022; Wei et al., 2022; Ahmad et al., 2023; Mikhaylova, 2023), detailed information on the specific criteria used by each country is often inaccurate. In the particular case of Paraguay’s regulatory framework, precise criteria guiding the regulatory status of products derived from NBTs remain elusive or erroneously construed in the current scientific literature. To enhance the public understanding of this topic and provide an additional perspective, we examine the regulations surrounding NBTs in Paraguay.

## Regulatory landscape

Different countries deploy diverse criteria to oversee crops derived from NBTs. Typically, these criteria consist of a definition, such as the Genetically Modified Organism (GMO) definition; triggers, like “novel trait” in the Canadian regulatory framework; or a roster of inclusions and exclusions, as utilized in the Australian regulatory system (Duensing et al., 2018).

Argentina was a pioneer in the development and application of regulatory guidelines for NBT crops in 2015, and since 2014 it has earned the distinction of “Reference Center for Biosafety” from the Food and Agriculture Organization (FAO) (Ministerio de Economía de Argentina, 2022). The country’s national biotechnology regulatory agency, CONABIA, provides guidance and technical assistance and engages in international cooperation to promote the development of sound regulatory frameworks and approaches for biotechnology.

The Argentinian model focuses on regulating NBT crops which involve permanent insertion of foreign DNA. In other words, products obtained through modern biotechnology techniques that do not have a new combination of genetic material are considered conventional (Goberna et al., 2022; Pixley et al., 2022). Several countries have adopted similar approaches based on Argentina’s lead (Table 1).

**TABLE 1:** Overview of various countries that have adopted the Argentinian criteria concerning New Breeding Techniques (NBTs).

Country	Party to the Cartagena protocol	NBTs first regulated (year)	Current NBT regulation	References
Argentina	No	2015	Resolution No. 21/2021 and three annexes^2^	Goberna et al. (2022)
Chile	No	2017	Consultation procedure^3^	Sanchez (2020)
Brazil	Yes	2018	Resolution No. 16/2018^4^	Nepomuceno et al. (2020)
Colombia	Yes	2018	Resolution No. 00022991^5^	Menz et al. (2020)
Paraguay	Yes	2019	Resolution MAG No. 842/2019^6^	Organization for Economic Cooperation and Development (2023b)
Ecuador	Yes	2019	Ministerial Agreement No. 063^7^	Gatica-Arias (2020)
Guatemala	Yes	2019	Resolution No. UA 60–2019: https://visar.maga.gob.gt/visar/2019/20/InMini60-19.pdf and Annex: 65.06.01:18^8^	Hernandez-Soto et al. (2021)
Honduras	Yes	2019	Agreement SENASA 008–2019^9^	Gatica-Arias (2020)
Philippines	Yes	2020	NCBP Resolution No. 001^10^	Entine et al. (2021)
Nigeria	Yes	2020	National Biosafety Guidelines on Gene Editing^11^	Jenkins et al. (2021)
Kenya	Yes	2022	Guidelines for determining the regulatory process of genome editing techniques in Kenya^12^	Sprink et al. (2022)

It is important to highlight that multiple regulatory agencies in different countries work closely together to discuss regulations for products derived from modern biotechnology (Organisation for Economic Cooperation and Development, 2023a). Given the evolution of technologies used in the genetic improvement of organisms for agricultural use, these guidelines are subject to periodic updates, resulting in variations in the dates in which they come into force that are reported by the different sources.

## Regional collaboration

The Southern Agricultural Council (CAS) was established in April 2003, and comprises the Ministries of Agriculture of Argentina, Bolivia, Brazil, Chile, Paraguay, and Uruguay. It aims to identify collaborative regional initiatives for short- and medium-term cooperation. To facilitate this process, CAS has established the Agricultural Policy Coordination Network, which includes the Directors of Agricultural Policies of each member country, as well as various technical-scientific groups. One such group is Technical Group 5 (GT5-CAS) on Public Policies on Biotechnology (Rocha-Salavarrieta, 2022).

Recognizing the significance of NBTs for agricultural development, the GT5-CAS stresses the importance of making science-based decisions to facilitate research and development while avoiding unnecessary trade barriers. In light of this, the said Ministries have declared that they would “seek to work together and with third countries to avoid unscientific barriers to trade in gene-edited agricultural products” (Consejo Agropecuario del Sur, 2018) and they have agreed to “promote the development and application of agricultural innovations that will enable our region to sustainably produce more and better food that is safe and nutritious for human consumption in order to contribute to food security and nutrition, ensuring access to such innovations for all producers, thus promoting economic and social development.” (Consejo Agropecuario del Sur, 2016). They also aim to enhance capacity-building efforts and encourage collaboration among countries for information exchange regarding product development and regulatory progress. This strong institutional support has contributed to technical and regulatory advancements for NBTs within the Southern Cone region (Rocha-Salavarrieta, 2022; Soerensen and Valdovinos, 2022).

## Paraguay NBT regulation

The regulatory system for agricultural biotechnology in Paraguay has made significant progress since its implementation by the Ministry of Agriculture and Livestock (MAG) in 1997. These advancements aim to establish a sound framework based on scientific criteria that include key concepts such as familiarity, history of safe use, substantial equivalence, transportability, and problem formulation (Fernández Ríos et al., 2018; Benítez Candia et al., 2020).

Specifically, in the case of NTBs, in 2019, MAG implemented a process to assess whether crops derived from these new technologies fall within the scope of the GMO regulation (Ministerio de Agricultura y Ganadería del Paraguay, 2019), as outlined in the Cartagena Protocol on Biosafety. The determination resulting from this procedure classified crops as either conventional or non-conventional (Organisation for Economic Cooperation and Development, 2023b). This classification is based on two main criteria: a) the utilization of genetic engineering techniques and b) the creation of a new combination of genetic material achieved through stable and simultaneous integration of nucleic acid sequences that form an identifiable genetic construct (Secretariat of the Convention on Biological Diversity, 2000).

**FIGURE 1:**
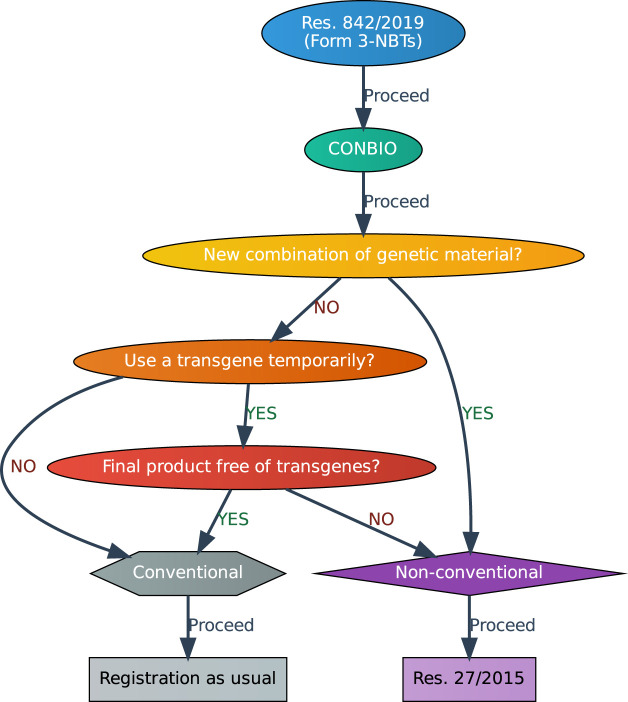
Decision-making flowchart that guides through various stages of determining whether a certain crop obtained through NBTs can be classified as “Conventional” or “Non-conventional” in Paraguay^13^. The process starts with the introduction of Form 3, leading to an analysis of the information presented by representatives of CONBIO. The primary determining factor is whether there is a “New combination of genetic material”.

The implemented procedure prioritizes a thorough evaluation on a case-by-case basis by the National Commission on Agricultural and Forestry Biosafety (CONBIO), free from any predetermined list of techniques or classification systems (e.g., SDN-induced variants). This procedure is not a risk assessment, and instead explores whether the NBT could result in genetic changes similar to those achieved through traditional breeding methods or naturally occurring ones. This norm gives certainty to developers about the regulatory status of a product. Furthermore, the analysis takes into account a science-based consideration of NBTs (Figure 1).

The application form consists of different sections, including: a) Applicant information, b) Organism information (taxonomy and the specific cultivar/line that will be introduced to the agroecosystem), c) Molecular biology details (description of the technique employed, target nucleotide sequences, and any functional modifications made; evidence demonstrating absence or presence of recombinant sequences), d) Phenotype considerations (examples of existing crops in the market with similar phenotypes, anticipated changes in proposed uses and management practices, analysis of the possibility of the occurrence of other effects beyond the desired phenotypes) and e) Authorizations (if the propagation material has been authorized by the official agency of any country) (Ministerio de Agricultura y Ganadería del Paraguay, 2019).

## Discussion

The regulatory landscape surrounding NBT crops on a global scale is dynamic, with countries continuously adapting their regulatory policies, which presents multiple opportunities not only to implementing countries but also at the regional level (Schmidt et al., 2020). This translates into a joint recognition that this set of techniques is important and relevant for the sustainable innovation of production systems, contributing to global food security and other societal benefits.

Some countries with established approaches to regulating NBT crops share the common practice of conducting analyses on a case-by-case basis and on novel combinations of genetic material as a threshold for regulation. According to this approach, the regulatory framework for NBT-derived crops is consistent with that of conventional breeding, if they are indistinguishable. Furthermore, this ensures that safety regulations prioritize traits, phenotypes, and intended uses (Friedrichs et al., 2019).

There is strong consensus that international regulatory coordination is needed to support the development of effective science-based regulatory systems for NBT crops, ensuring consistent and comprehensive oversight of these innovative technologies (Entine et al., 2021).

## Scope statement

The following manuscript is a scholarly contribution to the field of Biosafety. It focuses on New Breeding Techniques (NBTs) and their regulatory frameworks, with a particular emphasis on Paraguay and its consistency with regional criteria. Furthermore, it investigates regional collaborative initiatives, procedural approaches in Paraguay, and the complexities of NBT regulations. This manuscript provides valuable insights into the broader global landscape of NBTs in agriculture.

## Footnotes

1 The term “New Breeding Techniques” (NBTs) has been introduced to encompass the emerging technologies that harness modern biotechnology to create genetic diversity. However, it should be noted that as a class, NBTs lack universally agreed upon definitions (Whelan and Lema, 2015). Previously, the following have been classified as NBTs: genome editing, epigenetic modification, agroinfiltration, cis/intra-genesis, grafting with GMO material, reverse breeding, RNA interference and gene drives. For the purposes of this study, the term “NBTs” has been employed as synonymous with the terms “precision breeding,” “new plant breeding techniques,” or “new genomic techniques.” (Rosado and Eriksson, 2022).

2 Ex-Ministerio de Agricultura, Ganadería y Pesca (2021). Resolución N° 21/2021 S/EX-2020–72811589- -APN-DGD#MAGYP Establece procedimiento para determinar alcance en el marco de la Resolución N° 763/2011 - Nuevas Técnicas de Mejoramiento. Available at: https://magyp.gob.ar/sitio/areas/biotecnologia/conabia/_pdf/Resolution_N21-2021_3%20annexes.pdf.

3 Servicio Agrícola y Ganadero de Chile (2017). Aplicabilidad de Resolución N° 1.523/2001 en material de propagación desarrollado por nuevas técnicas de fitomejoramiento. Available at: https://www.sag.gob.cl/ambitos-de-accion/aplicabilidad-de-resolucion-ndeg-15232001-en-material-de-propagacion-desarrollado-por-nuevas-tecnicas-de-fitomejoramiento.

4 Comissão Técnica Nacional de Biossegurança, Brasil (2018). Resolução Normativa N° 16, de 15 de janeiro de 2018. Available at: http://ctnbio.mctic.gov.br/resolucoes-normativas/-/asset_publisher/OgW431Rs9dQ6/content/resolucao-normativa-n%C2%BA-16-de-15-de-janeiro-de-2018?redirect=http%3A%2F%2Fctnbio.mctic.gov.br%2Fresolucoes-normativas%3Fp_p_id%3D101_INSTANCE_OgW431Rs9dQ6%26p_p_lifecycle%3D0%26p_p_state%3Dnormal%26p_p_mode%3Dview%26p_p_col_id%3Dcolumn-2%26p_p_col_count%3D3%26_101_INSTANCE_OgW431Rs9dQ6_advancedSearch%3Dfalse%26_101_INSTANCE_OgW431Rs9dQ6_keywords%3D%26_101_INSTANCE_OgW431Rs9dQ6_delta%3D15%26p_r_p_564233524_resetCur%3Dfalse%26_101_INSTANCE_OgW431Rs9dQ6_cur%3D2%26_101_INSTANCE_OgW431Rs9dQ6_andOperator%3Dtrue.

5 Instituto Colombiano Agropecuario (2022). Resolución No. 00022991. Available at: https://www.ica.gov.co/getattachment/e4b3a97e-b44e-4974-8bb0-f3b947063e67/2022R22991.aspx.

6 Ministerio de Agricultura y Ganadería de Paraguay (2019). Resolución MAG N° 842/2019 Por la cual se aprueba el Formulario 3 de consulta previa para productos obtenidos mediante Nuevas Técnicas de Mejoramiento (New Breeding Techniques). Available at: https://conbio.mag.gov.py/media/ckfinder/files/RES.N842%2010%20DE%20JULIO%20DE%202019%20NBT.pdf.

7 Ministerio de Agricultura y Ganadería de Ecuador (2023). Ministerial Agreement No. 063. Available at: http://servicios.agricultura.gob.ec/mag01/pdfs/aministerial/2023/063-2023_acuerdo_ministerial_guia_tecnica_edicion_genetica_vf_numerado-signed.pdf. Article 401 of The Constitution of Ecuador, as established in 2018, declares that the country is free of transgenic crops and seeds. Although the presence of transgenic crops or seeds in Ecuador is limited, consumption of food from GMOs or their derivatives is permitted as long as proper labeling is provided. However, regulations specific to NBTs are not included in these provisions. Organisms that are genetically improved through genome editing or other NBTs will not undergo risk assessment if foreign or recombinant DNA is not present in the resulting improved organism. Ministerial Agreement No. 063/2023 is consistent with the existing regulatory framework for genetically modified organisms because it contributes to the procedures for determining whether a seed or crop contains foreign genetic material and whether it is part of the seed or crop genome.

8 Ministerio de Agricultura, Ganadería y Alimentación de Guatemala (2019). Resolution No. UA 60-2019. Available at: https://visar.maga.gob.gt/visar/2019/20/InMini60-19.pdf.

9 Servicio Nacional de Sanidad e Inocuidad Agroalimentaria de Honduras (2019). Acuerdo C.D. SENASA 008–2019. Available at: https://senasa.gob.hn/web/wp-content/uploads/2022/02/ACUERDO-CD-SENASA-008-2019-GACETA-35047.pdf.

10 National Committee on Biosafety of the Philippines (2020). Resolution No. 001. Available at: https://bch.dost.gov.ph/downloads/category/8-the-regulation-of-plant-and-plant-products-derived-from-the-use-of-plant-breeding-innovations-pbis-or-new-plant-breeding-techniques-nbts?download=23:pbi-resolution.

11 National Biosafety Management Agency, Federal Republic of Nigeria (2020). National Biosafety Guidelines on Gene Editing. Available at: https://nbma.gov.ng/wp-content/uploads/2022/03/NATIONAL-GENE-EDITING-GUIDELINE.pdf.

12 National Biosafety Authority, Kenya (2022). Guidelines for determining the regulatory process of genome editing techniques in Kenya. Available at: https://www.biosafetykenya.go.ke/images/GENOME-EDITING-GUIDELINES-FINAL-VERSION-25th-Feb-2022-03.pdf.

13 Ministerio de Agricultura y Ganadería (2015). Resolución MAG No 27/2015 Por la cual se aprueban los documentos Formulario 1: Ensayos regulados, 2: Liberación comercial y de la guía para Formulario 2: Liberación comercial Available at: https://conbio.mag.gov.py/media/ckfinder/files/Resolucion%20MAG%20N%2027_15%20Formularios_1.PDF (Accessed November 3, 2023).
